# Antitumor effect of Iso-mukaadial acetate on MCF-7 breast cancer mice xenograft model

**DOI:** 10.1038/s41598-024-64474-x

**Published:** 2024-06-14

**Authors:** P. P. Raphela-Choma, R. Lukhwareni, M. B. C. Simelane, L. R. Motadi, M. S. Choene

**Affiliations:** Department of Biochemistry, University of Johannesburg, Corner Kingsway and University Road, Auckland Park, Johannesburg, 2092 South Africa

**Keywords:** Breast cancer, MCF-7, Antitumor, Iso-mukaadial acetate, Apoptosis, Athymic-nude mice, Biochemistry, Cancer, Molecular biology

## Abstract

Antitumor drugs used today have shown significant efficacy and are derived from natural products such as plants. Iso-mukaadial acetate (IMA) has previously been shown to possess anticancer properties by inducing apoptosis. The purpose of this study was to investigate the therapeutic effect of IMA in the breast cancer xenograft mice model. Female athymic nude mice were used and inoculated with breast cancer cells subcutaneously. Untreated group one served as a negative control and positive control group two (cisplatin) was administered intravenously. IMA was administered orally to group three (100 mg/kg) and group four (300 mg/kg). Blood was collected (70 μL) from the tail vein on day zero, day one and day three. Tumor regression was measured every second day and body mass was recorded each day. Estimation of serum parameters for renal indices was examined using a creatinine assay. Histopathological analysis was conducted to evaluate morphological changes of liver, kidney, and spleen tissues before and after compound administration under a fluorescence light microscope. Histopathological analysis of tumors was conducted before and after compound administration. Apoptotic analysis using the TUNEL system was conducted on liver, kidney, and spleen tissues. Tumor shrinkage and reduction in body mass were observed after treatment with IMA. Serum creatinine was slightly elevated after treatment with IMA at a dosage of 100 and 300 mg/kg. Histopathological results of the liver exhibited no changes before and after IMA while the kidney and spleen tissues showed changes in the cellular structure. IMA showed no cytotoxic effect on the tumor cells, and cell proliferation was observed. Apoptotic assay stain with TUNEL showed apoptotic cells in spleen tissue and kidney but no apoptotic cells were observed in liver tissue section treated with IMA. IMA showed clinical toxic signs that resulted in the suffering and death of the mice immediately after IMA administration. Histopathology of tumor cells showed that IMA did not inhibit cell proliferation and no cellular damage was observed. Therefore, based on the results obtained, we cannot make any definitive conclusion on the complete effect of IMA in vivo. IMA is toxic, poorly soluble, and not safe to use in animal studies. The objective of the study was not achieved, and the hypothesis was rejected.

## Introduction

Breast cancer is the most common cancer and cause of leading death in women worldwide. It is also reported to be one of the most common types of cancer in South African women^1^. It is well known that breast cancer develops due to genetic mutations^2^. Surgery, radiation therapy, chemotherapy, endocrine therapy, and targeted therapy are some of the possible treatments for breast cancer patients. The subtype of breast cancer and the stage upon diagnosis must be taken into consideration while choosing a treatment^3^. The drawback is that the treatments are expensive therefore, cost-effective, easily accessible treatment is needed to accommodate those who cannot afford it^4^.

The anticancer effect of medicinal plants has been reported to inhibit cancer growth and proliferation and induce cell death via apoptosis^5^. They possess many beneficial properties such as high efficacy and low toxic effects^6^. It has been reported that some natural products or compounds have better ligands for biological targets as compared to synthesized compounds^7^. Iso-mukaadial acetate (IMA) exhibited anticancer activity on several cancer cell lines and has been exhibited to induce apoptosis using different mechanisms depending on the cancer cell line. This study aims to investigate the therapeutic effect of IMA on human breast tumor nude-xenografted mice models.

## Methodology and materials

### Isolation of *Iso*-mukaadial acetate from *Warbugia salutaris* stem bark

Iso-mukaadial acetate was isolated from *WS* stem bark^8^. The stem bark was obtained from KwaZulu Natal, botanical garden, South Africa (29.6079° S, 30.3478° E). The stem bark was sun-dried for 3 weeks (due to the season the bark was collected). The stem bark was crushed into finer particles using mortar and pestle. The starting material weighed 127,729 g. The powdered form of the stem bark was exposed to the maceration technique with dichloromethane for 3 days at room temperature (25 °C). The mixture was also mechanically shaken periodically. Crude extract weighed 79,778 g before subjecting it to the process of isolation of the compound of interest. The crude extract was filtered through Whatman (no 1), and the pressure was measured using water bath B-480 at 45 °C. Silica gel column chromatography with the following dimensions was used (60 × 1000 mm). Silica gel 60: 0.063–0.200 mm, was purchased from Merck and the column chromatography was designed and purchased from the Department of Chemistry (UJ).

The column was used to isolate the known compound of interest. Hexane and ethyl acetate (with different polarities) were used for the isolation of the compound. The compound was eluted at a 2:8 ratio (20% ethyl acetate and 80% hexane). Thin-layer chromatography techniques were used to identify the compound with the known standard compound. Sulphuric acid (10%) and distilled water (90%) were used to visualize the spots on the TLC plate by immersing the plates in the solution and heat to expose the spots. About 3, 09 g of the compound was isolated, and the chemical identification of the compound was checked using Nuclear Magnetic Resonance (NMR) spectroscopy (see the Appendix).

### *Iso*-mukaadial stock preparation

Iso-mukaadial acetate was dissolved in 10% ethanol, 90% saline, and pH 4.58 for the preparation of a 300 mg/kg dose. For the preparation of low dosage (100 mg/kg), IMA was dissolved in 5% ethanol, 95% saline, pH 5.40. The stock concentrations were stored at 4 °C until further use.

### Ethical statement

This is a statement to confirm that all experimental protocols were approved by the North-West University (Potchefstroom), ethical number (NWU-00254-17-A5). This study was reported following the Animal Research: Reporting of In Vivo Experiments (ARRIVE) guidelines.

To avoid any possible biases to occur, after inoculation, the mice were randomly and equally divided into four groups, so that each group contained three female mice. After the tumor reached a size of 100 mm^3^, the IMA treatments and cisplatin therapy commenced. When the tumor reached a palpable size, that specific day was assigned as day zero. Group one served as a negative control group (no treatments) and group two was the positive control group and received a known anticancer drug, cisplatin (4.5 mg/kg), which was administered intravenously (IV). The IMA treatment was administered orally to group three (100 mg/kg) and group four (300 mg/kg), which act as the treatment groups. Oral administration was done daily, and cisplatin was administered once on day 1. The duration of the study was supposed to be 14 days, but it ended up being 4 days due to the health condition of the animals after IMA administration.

### Animals

Female athymic nude mice (N = 12), aged 6–8 weeks (18–22 g) bred at the PCDDP Vivarium were used. There were four experimental groups, each containing 3 animals (n = 3). All mice were given a unique identification number according to SOP_Viv_Anim 5: Mouse numbering. They were housed in enriched Individually Ventilated Cages (IVC) Rack Isolator System (equipped with input and exhaust fan filter units that provide HEPA-filtered inlet and outlet air) within the PCDDP Vivarium at the Potchefstroom campus of the North–West University. Room temperature of 22 ± 1 °C, relative humidity of 55% (± 10%), a light/dark cycle of 12 h, and ventilation of 20 air changes per hour under positive pressure where the conditions mice were put in. Animals were provided with water ad libitum-fed standard rodent maintenance chow and housed on bedding derived from dust-free and non-toxic exfoliated corncob chips to absorb urine, excessive moistness, and potentially hazardous ammonia vapors.

### Sample size

Previous studies have shown that between six and eight animals per study group will be sufficient for tumor-based xenograft studies^9–13^. In this study, 12 mice developed tumors and the last 12 mice did not develop tumors or developed edema and died.

### Cell culturing

The human breast cancer cell line, MCF-7 (purchased from ATCC), was cultured in Dulbecco’s Modified Eagle Medium (DMEM), supplemented with 10% fetal bovine serum (FBS), 1% antibiotics (100 U/ml penicillin & 100 µg/ml streptomycin), 1% antimycotic (250 µg/ml amphotericin B), 1% non-essential amino acid solution (NEAA), and 2 mM l-glutamine. The cells were incubated in T-75 tissue culture flasks at 37 °C in a humidified atmosphere of 5% carbon dioxide. Every second day the media was changed if needed and cells were observed for confluency. Trypsin was used to detach the cells once the cells reached 80% confluence, after which the cells were harvested and counted for the xenograft study.

### MCF-7 inoculation in athymic nude mice

The inoculation was conducted according to the Establishment of Tumor-Bearing Rodent Models (Ethics number NWU-00254-17-A5) protocol. One million (1 × 10^6^) viable MCF-7 cells in a 0.1 µl Matrigel cell suspension were then subcutaneously injected into the right hind leg after the animals had undergone 5 days of acclimatization. An oestradiol supplement (4 mg/kg) was given once a week. The health of all the animals was monitored daily according to the SOP_Viv_Anim 27: Tumor growth was also measured every second day.

The tumor volume was measured with a linear digital caliper and was recorded every second day. The tumor volume was calculated using the equation below:

$${\text{Tumor volume }} = {\text{ W2 }} \times {\text{ L}}/{2}$$

W represents tumor width, measured from its widest point and L represents tumor length measured from its longest point. The relative tumor volume (RTV) was determined based on the method described by^12^. Accordingly, RTV was calculated using the formula (RTV = Vn/V0), where Vn is the tumor volume measured on a corresponding day, and V0 is the tumor volume measured at day 0. Each animal was weighed at the time of treatment to adjust the daily dose of the treatments. After treatment, the general health condition and clinical signs of the mice were monitored according to SOP_Viv_Anim 27: Determining pain and distress, for the duration of the study and recorded the observations.

### Sample collection and euthanasia

Blood was collected (70 µL) from the tail vein on day zero, and before euthanization (1 ml for terminal blood collection). After collection of blood, the blood samples were centrifuged at a speed of 2000×*g*, a temperature of 4 °C for 10 min to separate plasma. The plasma supernatant was stored at 20 °C until further analysis. All animals were euthanized according to SOP_Viv_Anim 1: Euthanasia proceeded through cervical dislocation.

*Note* The 5 animals of the test compound were euthanized after a short period of monitoring them due to the moribund state of the animals. The organs of the remaining animals were harvested (kidney, liver, and spleen) and the tumors were collected and stored accordingly.

### Estimation of serum parameters for renal indices

The estimation of serum parameters was set up as described below.

**Table:** 

Reagent	Sample blank (µl)	Standards (µl)	Samples
Creatinine assay buffer	44	The final volume of 50 µl	37
Creatinase	2	2	2
Creatininase	–	2	2
Creatinine enzyme mix	2	2	2
Creatinine probe	2	2	2
Standard	–	0, 2, 4, 6, 8, 10 nmol/well standard	
Sample	–	–	5

A final volume of 50 µl was added into the wells of a black plate. The reaction ix was put on a horizontal shaker to mix well and was incubated for 60 min at 37 °C away from light. Serum creatinine levels were analyzed or measured using absorbance reading at A_570_ nm. The blank was subtracted from the samples to obtain the correct measurements of creatinine level in the serum before and after treatment.

### Histopathological analysis of IMA before and after treatment

The organ samples were fixed in 10% neutral buffered formalin. To start the procedure of histopathology, the fixed samples were subjected to distilled water to wash off the formalin in the samples for 2 h. The samples were then dehydrated in rising ethanol for 45 min each (30%, 50%, 70%, 80%, 90%, 95%, and 2X 100%). To clear the samples, 2 replacement 100% xylene were used (10 min) and the infiltration step was done with 4 replacements of paraffin at 60 °C. See below:

#### Infiltration step

**Table:** 

1:1 Xylene:	Wax for 30 min
Pure wax	1 h
Pure wax	2 h
Pure wax	Overnight

Then the samples were embedded in paraffin wax blocks. A warm tray, forceps, and a lit candle were used. The warm metal blocks were taken out of the oven and applied Vaseline on them. An L-shaped steels were used to make a block to pour in the wax then immediately immersed a tissue. To make sure that the tissue was properly positioned, we continued adding the wax, evened it out using a spatula, and removed bubbles with a heated needle. Covered the tray with water so that the wax may hold firmly, then the set block was removed, labeled, and stored at 4 °C. Then the samples were sectioned using a manual rotary microtome at 5 µm. A water bath was used to place in the sectioned tissues in and were transferred on a microscope slide and stored for further analysis.

#### Hematoxylin and eosin staining

The procedure was as follows for hematoxylin and eosin staining:

**Table:** 

Step 1: Xylene × 2	5 min
Absolute ethanol × 2	5 s
Running tap water	2 min
Hematoxylin	8 min
Running tap water	Rinse
Scott’s tap water	3 min
Running tap water	8 min
70% ethanol	1 min
Eosin Y	90 s
Running tap water	Rinse
Absolute ethanol × 2	5 s
Last step: Xylene × 2	5 min

The stained-glass slides were taken out and Dibutylphthalate Polystyrene Xylene (DPX) mountant was used to mount the coverslips on the glass slides then the slides were viewed by Zeiss Axiovision fluorescence light microscope. Images were captured.

### Colorimetric apoptotic assay using TUNEL system

The following procedure was conducted using manufacture’s protocol (Promega).

#### Pretreatment procedure of tissue section

**Table:** 

	Washed with xylene	5 min
2nd wash	100% ethanol	5 min
Rehydration	100, 95, 85, 70 and 50% ethanol	3 min each
3rd Wash	0.85% NaCl	5 min
4th Wash	1Xpbs	5 min

#### Detection of apoptotic

After the pretreatment of the slides, they were fixed by immersing them in 4% paraformaldehyde dissolved in Phosphate buffer saline (PBS) for 15 min. The slides were washed with PBS by immersing the slides in the solution for 5 min (twice). To permeabilize the tissue samples, proteinase K solution was added, and the slides were incubated at room temperature for 20 min. The slides were washed with PBS for 5 min after incubation. The fixation step was repeated, immersed the slides in 4% paraformaldehyde solution for 5 min and washed with PBS for 5 min. An equilibration buffer was added and equilibrated at room temperature for 10 min. The Terminal deoxynucleotidyl transferase (TdT) reaction mix was added to the tissue section on the slides and coverslips were used to avoid complete dryness of the tissues. The tissue slides were incubated for 60 min at 37 °C. To stop the reaction, coverslips were removed, and the slides were immersed in 2 × saline sodium citrate (SCC) for 15 min. The slides were washed three times with PBS for 5 min each wash. Hydrogen peroxide (0.3%) was used to block the reaction for 5 min and washed again three times with PBS for 5 min each wash. Streptavidin Horseradish Peroxidase (HRP) diluted in PBS was added and slides were incubated at room temperature for 30 min. The slides were washed after incubation three times with PBS for 5 min each wash. For staining, DAB solution was prepared and developed until a light brown color was observed and was added on the slides. Deionized water was used to wash the slides and slides were mounted with a permanent mounting medium. The slides were viewed and analyzed under a fluorescence light microscope.

### Statistical analysis

All protocols, reports, and raw and analyzed data are the property of the sponsor but will be stored at the PCDDP as described in the SOP_QA_Arch 1: Archiving of documents. The documents will be archived for 15 years. Statistical analysis of the results was performed using Graph Pad Prism and Zeiss Axiovision software. The data were expressed as mean ± Standard Deviation (*P < 0.05, **P < 0.01 ***P < 0.001). A *P*-value less than 0.05 (*P* < 0.05) was considered significant.

## Results

### Summary of the in vivo results

**Figure 1:**
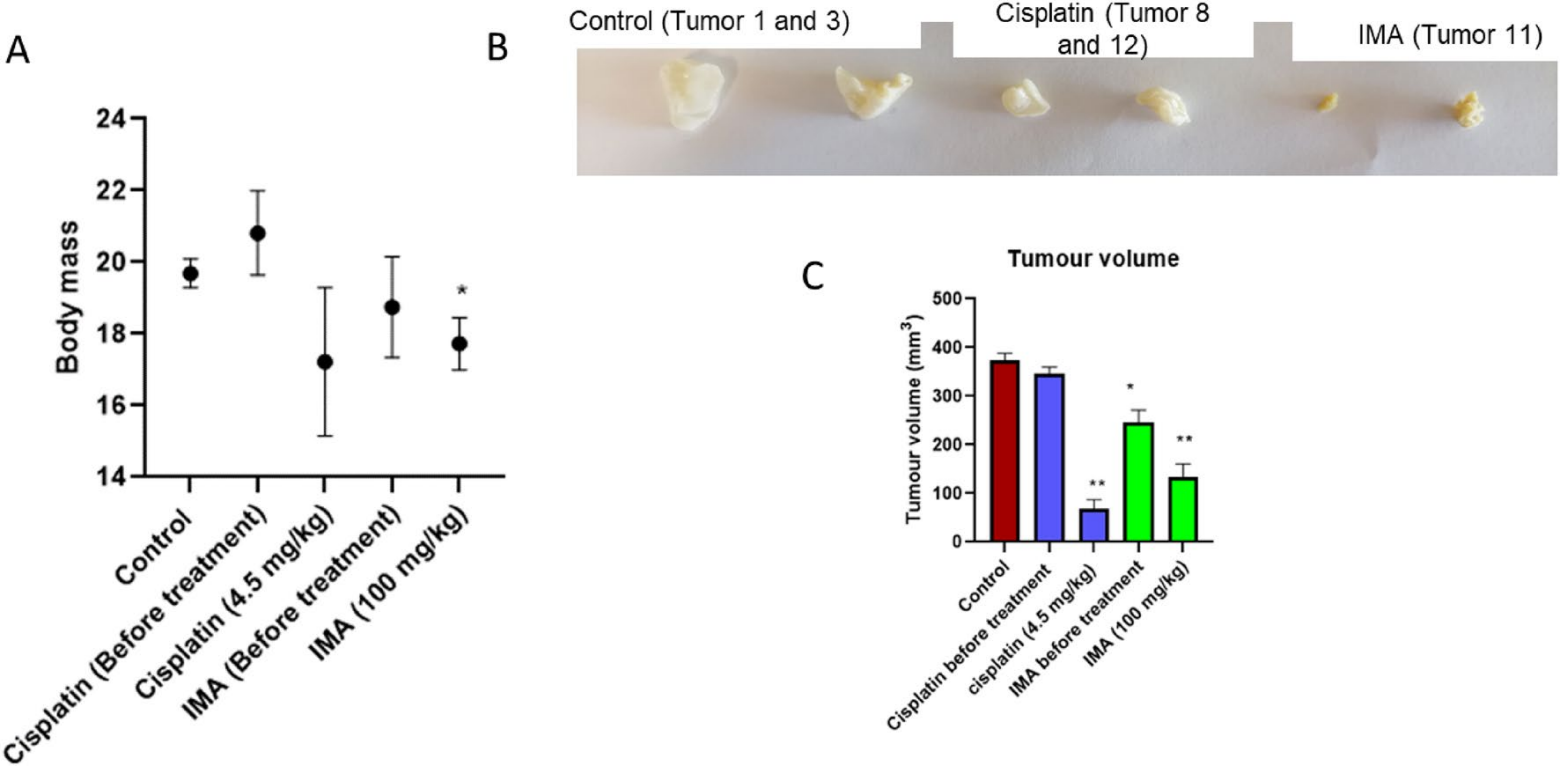
The body mass and tumor volumes before and after treatment with a low dose of IMA (100 mg/kg) and positive control (4.5 mg/kg) cisplatin were measured. (**A**) shows the change in body mass of the treated mice. (**B**) and (**C**) Show tumor growth and volume of the control group, positive control-treated group, and IMA-treated group. The data from two biological replicates were expressed as mean Standard Deviation (*P 0.05, and **P 0.01).

**Figure 2:**
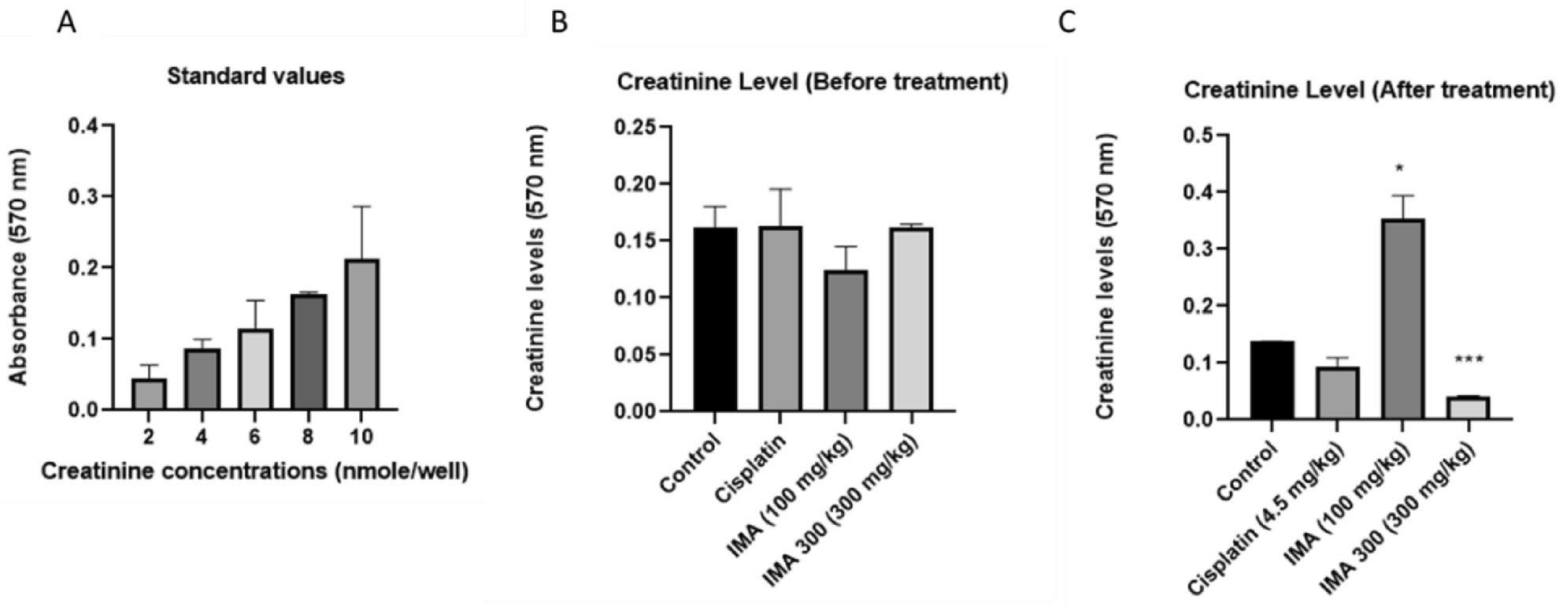
Serum creatinine was measured to assess renal function in control and treated mice with cisplatin (4.5 mg/kg) and IMA (100 mg/kg). The levels of creatinine in the serum were determined. There was a significant change in the level of serum creatinine after treatment (**C**), cisplatin was lower in comparison with control and IMA was slightly elevated in comparison with control. The data from two biological replicates were expressed as mean Standard Deviation (*P 0.05, and ***P 0.001).

**Figure 3:**
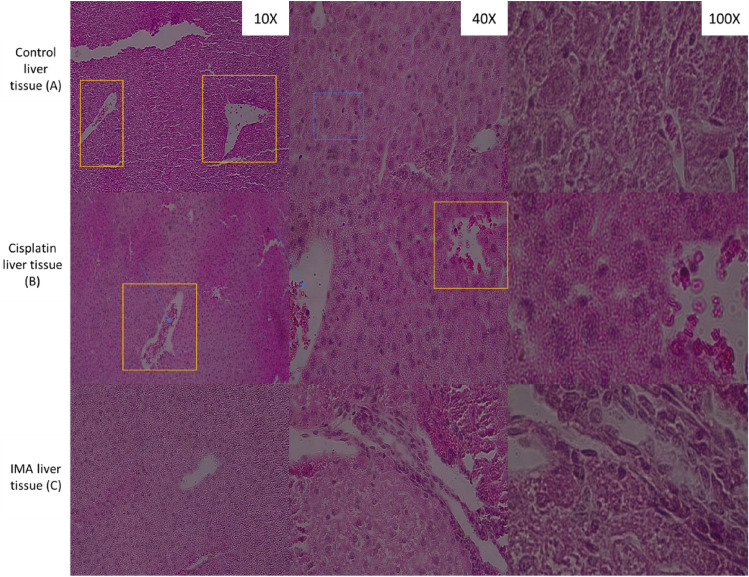
Histopathological observation of liver tissue from control mice group (**A**), Cisplatin group (**B**), and IMA group (100 mg/kg) (**C**) stained with Hematoxylin and Eosin dyes. Orange square boxes show central veins, portal, and sub-lobular veins (**A**, **B** and **C**). Blue arrows show erythrocytes and the blue box shows granular cytoplasm. The same size, and shape of cells and endothelial cells were observed. No distinction of cellular structures was observed. A fluorescence light microscope was used to analyze the results with 10× , 40× , and 100× magnification.

**Figure 4:**
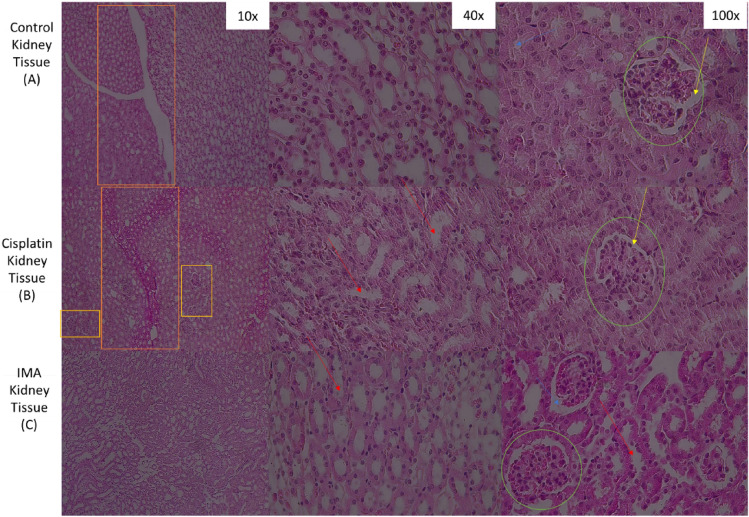
Histopathological observation of kidney tissue from control mice group (**A**), Cisplatin group (**B**) and IMA group (100 mg/kg) (**C**) stained with Hematoxylin and Eosin dyes. There were some distinctions observed before and after treatment with IMA and cisplatin. Control kidney section showed normal structure of glomerulus, bowman’s capsule (green circle, yellow arrows) and renal tubules (blue arrow). Cisplatin tissue section (**B**) showed proximal tubule and renal capsule (orange box). A red arrow showed renal tubules on IMA treated (**C**) tissue section. A fluorescence light microscope was used to analyze the results with 10× , 40× and 100× magnification.

**Figure 5:**
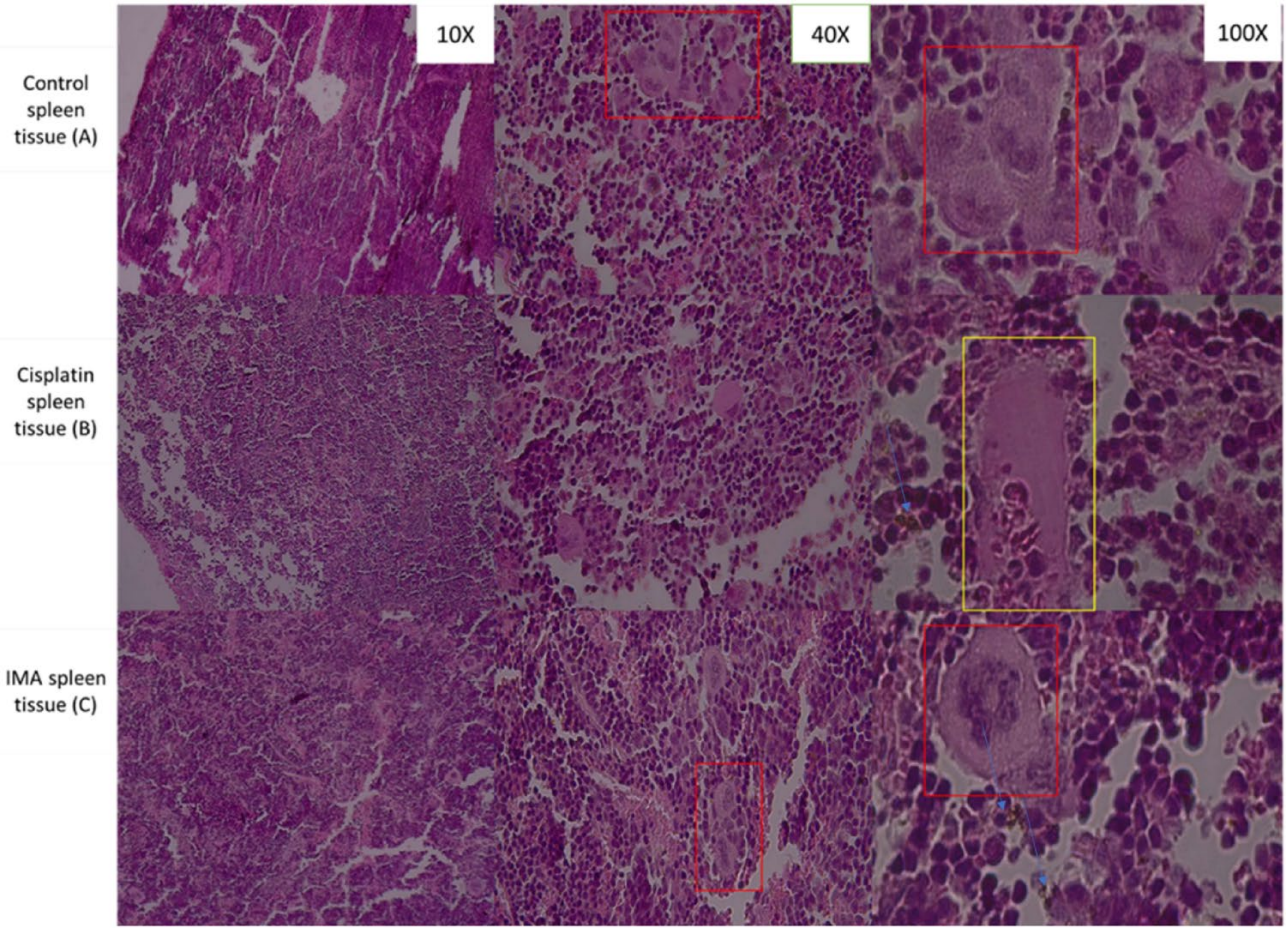
Histopathological observation of spleen tissue from control mice group (**A**), Cisplatin group (**B**) and IMA group (100 mg/kg) (**C**) stained with Hematoxylin and Eosin dyes. Red box is white pulp with red pulp in the middle. Yellow box shows vein with erythrocytes. Blue arrows show a brown pigmentation which is hemosiderin. A fluorescence light microscope was used to analyze the results with 10× , 40× and 100× magnification.

**Figure 6:**
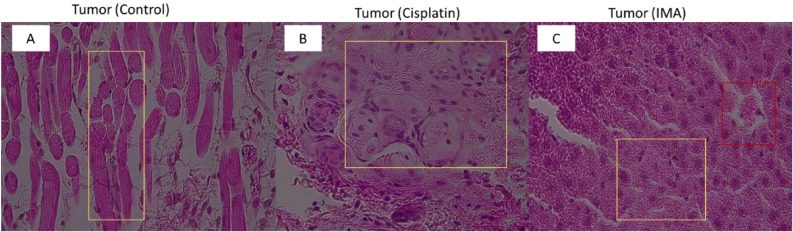
Histopathological observation of tumors from control mice group (**A**), Cisplatin group (**B**) and IMA group (100 mg/kg) (**C**) on day 4. Hematoxylin and Eosin stains were exposed to tissue slides. Control group (**A**) showed skeletal muscle tumor cells (yellow box), positive control cisplatin (**B**) showed well-defined cell structure with cancer cell growth (Yellow box) and IMA (**C**) showed no toxicity effect on the cancer cell (yellow box). The red box (C) showed erythrocytes within the arteries. A fluorescence light microscope was used to analyze the results with 40× magnification.

**Figure 7:**
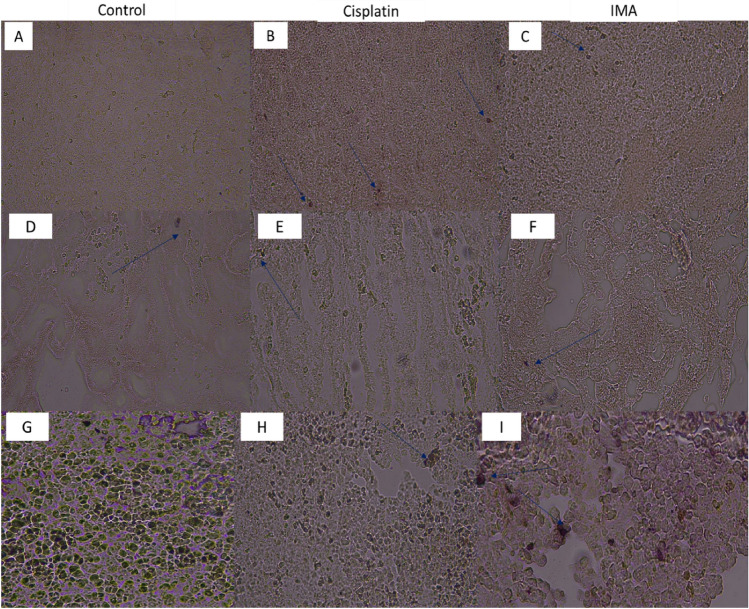
Detection of apoptosis on liver, kidney, and spleen tissues stained with TUNEL dye. The control group (**A**, **D**, and **G**) showed no cells that were undergoing apoptosis in all three tissues. The Cisplatin group (**B**) and IMA group showed cells that underwent apoptosis (blue arrows). The intensity of the staining was low IMA in comparison to cisplatin which means the apoptotic cells were minimal. Cisplatin group (**E**) and IMA group (**F**) showed very few cells that may have undergone apoptosis (blue arrows) in the kidneys. The intensity of the staining was very low in all groups which may show that the cellular death process was at the earliest stage. The Cisplatin group (**H**) and IMA group (**I**) showed minimal cells that may have undergone apoptosis (blue arrows). The intensity of the staining was high in IMA (**I**) in comparison to cisplatin. A fluorescence light microscope was used to analyze the tissue-stained slides at 40× magnification.

Following inoculation and observation of tumor growth, only 8 mice developed tumors to the size of 100 mm^3^ for the commencement of the treatment. However, through the treatment the following groups were continued, group 1 with 3 mice, group 2 with 2 mice, and group 3 with 1 mouse. The results represent the nude xenografted mice’s progress following inoculation and treatment with IMA as well as the positive control cisplatin. The body mass of all mice in this study was taken as well as the tumor volume (Tables 1 and 2, and Fig. 1). Renal function was determined using the plasma of all the mice including group 4 (Fig. 2). The potential mechanism of tumor growth inhibition of human breast nude xenografted mice was determined by hematoxylin and eosin stain and (terminal deoxynucleotidyl transferase biotin-dUTP nick end labeling) TUNEL staining before and after treatment with IMA and cisplatin (positive control). These results showed that the administration of IMA had an immediate effect hence the state of the mice after IMA dosage (100 mg/kg) (Figs. 3, 4, 5, and 7). Lastly, the tumor tissues were analyzed using the hematoxylin and eosin stains (Fig. 6). The tumor cells showed well-defined cellular structures and no sign of apoptosis or cellular damage after treatment with IMA in comparison to the control group.

**Table 1:**
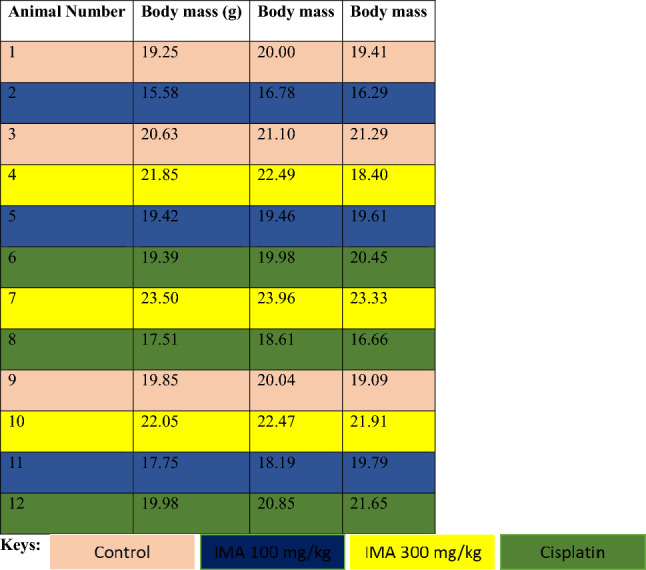
The results below show the body mass results of all mice groups before treatment with IMA. In the control group 1, there was a loss in body mass of about 0.59 g the day before treatment. Mouse 3 was growing well with an increase of 0.1 body mass. Group 2 (cisplatin) showed that mice 6 and 9 were growing well, with an increase in body mass. While mouse 8 lost about 1.95 g of body mass before treatment. Mice 5 and 11 in group 3 (100 mg/kg) were growing well as there was an increase in body mass before treatment with IMA. Mouse 5 gained 0.15 g whereas mouse 11 gained 1.6 g. Mouse 2 showed a decrease in body mass the day before treatment by 0.49 g. The last group 4 (300 mg/kg) also showed a drastic decrease in body mass for mouse 4 before treatment (4.09 g) while mice 7 and 10 mass showed a slight decrease by 0.63 g and 0.58 g.

**Table 2:**
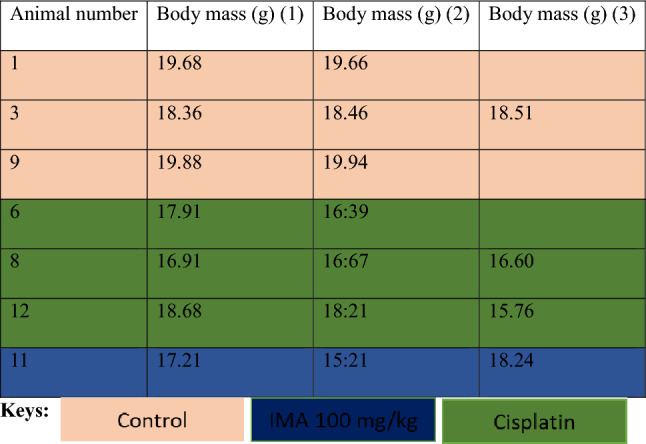
The results below show the body mass of the mice after treatment with IMA. There was an increase in body mass of mice 3 and 9 in the control group while mouse 1 slightly decreased by 0.02 g. Group 2 (cisplatin) showed a drastic decrease in body mass after treatment. Group 3 (IMA) showed a decrease in body mass (2 g) after treatment and a drastic increase in body mass (3.03 g) on day 4 before euthanasia.

### The body mass of the animals (last 3 days body mass before treatment)

See Table 1.

### The body mass of the animals after treatment

See Table 2.

### Summary of the analysis of body mass and tumor volume following treatment with IMA

The results above compare the change in body mass before (Table 1, Fig. 1A) and after treatment (Table 2, Fig. 1A) in comparison with the control group. The average body mass of the nude mouse used was around 19.00–23.00 g according to the results. The mice treated with IMA showed a significant body mass loss of 0.54 g. They also indicated a reduction in food and water intake. This was observed due to the toxic effect of IMA which affected the mobility of the mouse. A change in body mass was also observed before and after treatment with cisplatin in comparison to the control group by approximately 4.6 g. The significant decrease in tumor volume after treatment (Fig. 1A and B) showed that IMA may caused a reduction by approximately 119 mm^3^. While cisplatin led to or a change in tumor size by approximately 233 mm^3^ in comparison to the control.

### Analysis of the renal function of IMA

The above results showed that serum creatinine was within the normal range before treatment with cisplatin and IMA in comparison with the standard values. Control group value of 0.15 nm was measured by absorbance (Fig. 5B and C) which showed that there was approximately 8 nmole creatinine concentration in the kidneys and this was normal as was compared with the standard serum creatinine values (Fig. 5A). This value in the control group did not change throughout the experiment as expected. In the positive control group, 0.15 nm was measured which showed that about 8 nmole creatinine concentration was present before treatment and IMA groups showed that there were about 6–8 nmole creatinine concentration before treatment with absorbance of 0.13 and 0.16 nm. The values were within the normal range in comparison to the standard curve and control group. However, after treatment with cisplatin, a slight reduction in creatinine level was observed (about 2–4 nmole concentration). The reduction was still within the normal range of creatinine levels in comparison to standard values. IMA at 300 mg/kg dosage showed a drastic reduction of creatinine level (about 1–2 nmole concentration), this was found to be low and explains the moribund state the mice were in immediately after high IMA dosage. IMA 100 mg/kg dosage showed a drastic increase in creatinine level by 0.22 nmole concentration. The absorbance measurement was 0.35 nm, and this was out of the normal range which may explain that there was kidney dysfunction due to the toxicity of IMA. These results above showed that there was a significant change of the creatinine level after treatment with IMA.

### Histopathology of liver tissue using a fluorescence light microscope

Microscopically, the organization of liver can be represented by three different schematics the classic (hepatic) lobule, the portal lobule, and the hepatic acinus. From our study, we observed that in the control group of mice, similar results and morphology were observed in cisplatin treated. The morphology of IMA treated liver even though was not transformed or showing any severe damage the structure does not look similar to the two control groups.

### Histopathology of kidney tissues using a fluorescence light microscope

Microscopically, the normal kidney at low power has a thin connective tissue capsule at the left with underlying renal cortex which contains a glomerulus and surrounding tubules with cuboidal epithelium. When observing the kidney in the mice study as shown above, one observed the normal glomerulus and renal tubules that are responsible for the filtration of blood. The renal tubules shown by the red arrow also showed no changes in the morphological structure before and after treatment suggesting that there was no effect on the renal tubule.

### Histopathology of spleen tissue using a fluorescence light microscope

In mice, the normal spleen consists of the white pulp embedded in the red pulp. In the white pulp, T and B lymphocytes form accumulations, the periarteriolar lymphatic sheaths and the follicles, located around intermediate-sized arterial vessels, the central arteries. From the observed spleen above, the IMA did not produce any significant change in the cellular architecture of the spleen. The cisplatin did not produce any additional damage to the spleen of the animals. The pathological changes in the spleen were observed in the form of distorted lymphoid and minimized lymphoid follicles.

### Histopathology of tumors using fluorescence light microscope

Morphologically, the tumor architecture was not different in all three groups, that is the control group, cisplatin, and IMA-treated groups. As shown above, there is no change in the cellular characteristics of the tumor in all groups. This however must be noted that this was a 4-day study which could not give us a statistical understanding of the effect of IMA on cancer cells.

### Apoptotic observation of liver, kidney and spleen tissues using TUNEL system

To understand or see if there might be any other cellular mechanism of damage to the liver, spleen, and kidney, a TUNEL assay was conducted which evaluates the morphological appearance of apoptosis on tissue sections. From the stained tissues as shown above. There were minimal apoptotic bodies as shown by the arrows in the micrographs. These minimal apoptotic bodies suggest that the treatment may not have induced apoptosis in all the treated mice.

## Discussion

These are the first findings of the antitumor effect of IMA in breast cancer mouse models. Previously, IMA showed anticancer activities on breast and ovarian cancer cells^14^. This was possible because it was a single-cell treatment with no interferences from the whole-body system. In this current study, we report the effect of IMA in xenograft mice, the groups were divided into 4, namely: the untreated group, the cisplatin group that served as our positive control, the 100 mg/kg IMA group, and the 300 mg/kg group.

After administering IMA, mouse 11 exhibited a significant decrease in tumor volume (Table 2 and Fig. 1) in comparison to the before-treatment values as well as the control group. This observation showed that IMA (mouse 11) may possess antitumor effects as inhibition of tumor growth and reduction was observed within 2 days of treatment in comparison to the control group. It may also mean that the tumor reduction was due to spontaneous tumor reduction based on the pathological results of the tumors in Fig. 6. Cisplatin is mostly used in several studies as a positive control and shows a therapeutic effect on cancer mouse models^15^. In this study, a low dosage of cisplatin was used, it was administered once and the exposure time was limited, hence the effect on tumor cells was not observed (Fig. 6).

Creatinine is an important indicator of kidney function. Elevated levels suggest that the kidney might have been damaged while the normal levels suggest that the kidney might be functioning optimally and too low might suggest muscle damage. The serum creatinine level Fig. 2C in mice treated with IMA (300 mg/kg) was lower in comparison with the untreated serum samples (Fig. 2B). The lower amount of serum creatinine detected in these mice indicated that the IMA may have severely decreased muscle activity^16^ of the mice and caused kidney failure or hyperfiltration of the glomerulus^17,18^. The nude mouse administered with IMA at 100 mg/kg showed that the serum creatinine level was drastically elevated after treatment in comparison to untreated results and the standard values (Fig. 2), however, it may be within the creatinine normal range according to Keppler et al.^19^. A slightly elevated serum creatinine level may indicate the occurrence of clinically significant kidney disease^20^, and such signs were observed immediately after IMA administration.

Histopathological results of liver tissue (Fig. 3) after H and E stain showed no distinct cellular structural abnormalities in all groups. The control group, cisplatin group, and IMA group have normal same-sized granular cytoplasm and nucleus. Blood vessels and endothelial cells were observed. This means that IMA did not have a cytotoxic effect on the liver tissue of the mouse as no damage was detected. The kidney tissue section (Fig. 4) showed no structural changes in comparison to the control group. However, IMA low dosage may have slightly affected the function of the kidneys due to the increased serum creatinine observed in Fig. 2. After IMA administration, the mouse was observed to be in distress and shock, this may explain the possibility of kidney damage. Histopathology of spleen tissue from the control group showed normal histopathology structure of both white and red pulps. The spleen tissue (Fig. 5) also showed the depositions of hemosiderin pigmentation in the red pulp. It is known that hemosiderin pigmentation may be a result of the destruction of red blood cells^21^. The same observation was seen in mice treated with cisplatin. Melano macrophage centers (MMC) were not observed in the white pulp which usually appears if there is a compromised immune function or toxins to be destroyed or engulfed.

Histopathological observation of the tumor section treated with IMA (Fig. 6) showed that there was cellular proliferation, and no abnormal cells were observed because of IMA exposure. IMA was administered once (on day 0); therefore, it is expected that no effect be observed, the mammalian system is complex, and some processes need to take place for the drug to reach the target site or even the tissues. The apoptotic results in Fig. 7A–C showed that minimal cell death may have occurred in mice treated with cisplatin in comparison with IMA. Apoptotic results in Fig. 7D–F showed that apoptosis may have been elicited due to the toxicity of IMA exposure. In Fig. 7G–I, the spleen tissue showed apoptotic cells stained brown. The cells undergoing apoptosis showed apoptotic characteristics such as nuclear condensation and irregular cellular shape. Apoptosis is a natural process that occurs to eliminate abnormal cells in the body. It is expected to see cells undergoing this process regardless of IMA treatment or not.

In conclusion, this study did not go according to plan. It was abruptly stopped due to ethical rules and regulations of animal studies. Therefore, based on the results obtained, we cannot make any definitive conclusion on the complete effect of IMA in vivo. However, IMA is toxic, poorly soluble in saline solution, DMSO and it is partially soluble in ethanol when a higher concentration of the compound is used. Poor solubility leads to poor absorption and bioavailability of the compound, which may render it ineffective. IMA showed clinical toxic signs that resulted in the death of the mice immediately after administration. In the future, a different route of administration or IMA derivatives may be considered on human breast cancer mouse models.

## Figures and Tables

**Figure 8:**
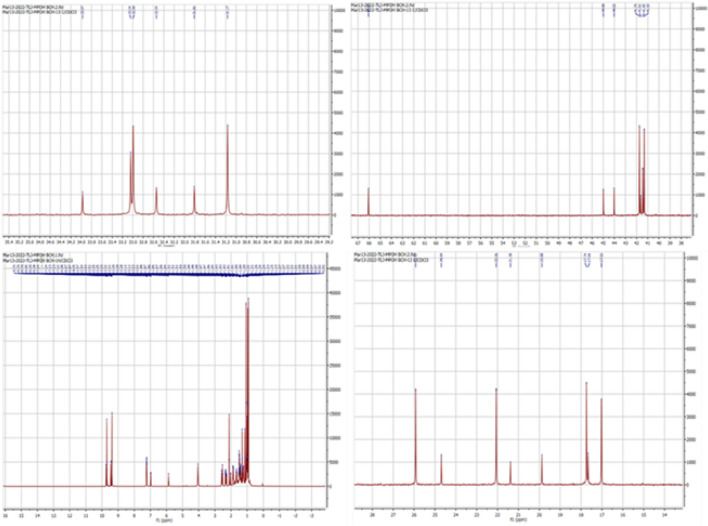
The chemical identification of IMA, and their unambiguous 1H, 13C assignments were achieved. The above results showed that the compound (IMA) isolated from *W. salutaris* was pure, and no impurities were detected.

## Data Availability

The datasets used and/or analyzed during the current study are available from the corresponding author on reasonable request.

## Appendix

See Fig. 8.
